# Reliability of preoperative evaluation of postmenopausal ovarian tumors

**DOI:** 10.1186/s13048-017-0309-4

**Published:** 2017-03-14

**Authors:** Riikka Johanna Niemi, Sami Kristian Saarelainen, Tiina Hannele Luukkaala, Johanna Unelma Mäenpää

**Affiliations:** 1 Department of Obstetrics and Gynecology, Tampere University Hospital, P.O. Box 2000, FI-33521 Tampere, Finland; 2 Research and Innovation Center, Tampere University Hospital and Faculty of Social Sciences, University of Tampere, FI-33014 Tampere, Finland; 3 Faculty of Medicine and Life Sciences, University of Tampere, FI-33014 Tampere, Finland

**Keywords:** Expert opinion, IOTA LR2, IOTA simple rules, Ovarian cancer, Ovarian tumor, Power doppler, Three-dimensional ultrasound

## Abstract

**Background:**

Preoperative evaluation of ovarian tumors is challenging. This study was undertaken to evaluate the performance of conventional two-dimensional (2D) ultrasound and CA125 in predicting malignant or benign nature of pelvic masses, and to investigate if three-dimensional power Doppler (3DPD) ultrasound provides any added value. Ninety-six postmenopausal and four perimenopausal women with supposed ovarian tumors were examined by standardized 2D and 3DPD ultrasounds preoperatively. The tumors were evaluated using the risk of malignancy index (RMI), International Ovarian Tumors Analysis (IOTA) group simple rules, expert opinion, IOTA logistic regression model 2 (LR2) and 3D vascular indices, and were postoperatively compared to histopathological results.

**Results:**

Ninety-eight tumors turned out to be ovarian in origin. Of these, 66 were benign and 32 malignant. RMI (cut-off value 200), simple rules, expert opinion and LR2 (cut-off value 25) were used to predict malignant nature of the tumors and had sensitivities of 71.9, 90.6, 87.5 and 90.6%, and specificities of 80.3, 84.6, 92.4 and 77.3%, respectively. When the 3D vascularization flow index (VFI) was added to RMI and LR2, the accuracy of the test improved from 77.6 to 81.4% and from 81.6 to 86.5%, respectively, at the expense of sensitivity, while VFI gave no added benefit for simple rules and expert opinion. Agreement between two examiners using expert opinion was good (Cohen’s kappa = 0.89).

**Conclusions:**

The subjective opinion of an expert seems to be the most reliable method in assessing ovarian tumors, and the 3DPD indices seem to provide no significant added value.

## Background

Ovarian cancer has the leading mortality rate of all gynecological cancers, and the incidence of ovarian cancer is at its peak among postmenopausal women. The optimal treatment of ovarian neoplasms requires correct preoperative characterization of tumors. The surgical treatment of ovarian cancer should be reserved for gynecological oncologists, while benign tumors can be operated on less radically by general gynecologists, or even managed conservatively.

Preoperatively ultrasound features combined with the measurements of CA125 has been used to predict the malignancy of a pelvis mass (Risk of Malignancy Index, RMI) since early 1990’s [1]. However, subjective assessment by an experienced ultrasound examiner has been considered to be the best diagnostic method for ovarian tumors [2, 3]. While not all gynecologists are so well accustomed to ultrasound examinations, in 2008 the International Ovarian Tumors Analysis (IOTA) group presented simple ultrasound-based rules that include five rules for predicting malignant tumors (M-rules) and five for predicting benign tumors (B-rules). If one or more M-rules with absence of B-rules or B-rules with absence of M-rules are present, the tumor is supposed to be malignant or benign, respectively. In a multicenter study, these rules were applicable for 76% of all tumors and showed a sensitivity of 95% and specificity of 91% [4]. If none of the rules is valid or if both M- and B-rules are present, a tumor is considered to be inconclusive [4, 5]. In that case the opinion of an expert ultrasound examiner is needed, which is called a two-step strategy [6]. In addition, the IOTA group developed two logistic regression models (LR1 and LR2) [7]. LR1 is based on 12 different variables, and LR2 is based on 6, including patient history, clinical signs and ultrasound features. These models have shown sensitivities of 92–95% and 89–95% with specificities of 74–87% and 73–86% in detecting ovarian malignancies, respectively [8]. Nevertheless, in several studies, the impression of an expert ultrasound examiner has still been considered to be the best method, or at least equivalent to LR1 and LR2, for diagnosing ovarian pathology [8, 9].

An increased density of microvessels and abnormal vascular tree of the tumor are characteristic of malignant ovarian processes [10, 11]. These phenomena have given new insight for the use of three-dimensional (3D) ultrasound and 3D power Doppler (3DPD) in evaluating the vascularization of ovarian tumors to discriminate between benign and malignant tumors. Unfortunately, there are no clear cut-off rules for 3D ultrasound features, limiting their clinical utility [12].

The purpose of this study was to examine if 3DPD ultrasound can offer additional benefits over conventional two-dimensional ultrasound and other diagnostic methods as a useful tool for predicting malignancy of an adnexal mass. The aim was to find the most applicable and reliable preoperative diagnostic approach for postmenopausal women.

## Methods

Between February 2011 and November 2014, one hundred women over 50 years of age presenting with an abnormal adnexal mass(es) at the Department of Obstetrics and Gynecology of Tampere University Hospital, were recruited to the study. All patients were destined for surgery. Overtly benign or malignant looking tumors like unilocular simple ovarian cysts and tumors associated with marked ascites (depth of the greatest pool over 10 cm) were excluded. In principle, the maximum allowed diameter of the tumors was 10 cm, allowing the entire tumor to be assessible transvaginally. However, there were five tumors, the maximum diameter of which exceeded 10 cm at the final examination, but it never exceeded 12 cm.

According to preoperative findings, the patients were operated on by either a gynecological oncologist or a general gynecologist, using either laparoscopic or open technique.

The patients were assessed within 2 weeks prior to surgery by vaginal two-dimensional (2D) and 3D ultrasound examination with power Doppler. In the case of bilateral ovarian tumors, both masses were examined, and the more complex tumor was assessed for the study. All ultrasound examinations were performed by an experienced gynecologist or the author R.J.N., using a Voluson 730 Expert unit (GE Medical Systems, Zipf, Austria) with a multifrequency transvaginal transducer (5–9 MHz). A normal B-mode ultrasound assessment included the calculation of the size of the adnexal mass. The power Doppler settings were standardized: frequency, 6 MHz; power Doppler gain, -0.6; wall motion filter (WMF), low 1; pulse repetition filter (PRF), 0.6 kHz. The extent of vascularization of the tumor was described by a score from 1 to 4 (1 = no blood flow detected, 4 = high blood flow detected) [13]. 3DPD was used to examine the ovarian tumor after the 2D evaluation. The acquisition sweep angle was set to 85°.

A serum sample was obtained preoperatively, and RMI was calculated by using the original formula of Jacobs et al. in which the serum CA125 level, ultrasound scan result and patient’s menopausal status are taken into account [1]. The ultrasound data were stored on a hard disk for later evaluation. Based on the ultrasound examination, the examiner classified the adnexal tumor as benign or malignant (expert opinion), immediately following the actual examination. From the 2D data, the tumors were classified by using the IOTA simple rules. If none of these rules applied or if both M- and B-rules were applicable, the tumor could not be classified. In such case, the tumor was evaluated subjectively by the examiner as benign or malignant.

The IOTA LR2 was implemented by using the Predictive IOTA models for ovarian cancer application software, version 2013 (App for IOS operating systems). In the LR2 model, the six variables used are age of the patient, presence/absence of ascites, presence/absence of papillations/papillary projections with blood flow, maximum diameter of the largest solid component, irregular cyst walls and presence/absence of acoustic shadows. This calculated model yields the probability of malignancy of an ovarian tumor.

The analyses of the stored 3D volumes were performed by the same investigator (R.J.N.) using a virtual organ computer-aided analysis (VOCAL™) imaging program and 4D-View software (GE Healthcare, v 9.1). The volume of each adnexal tumor was measured by manual delineation of the contour of the mass with a 15°-rotation step. Using the VOCAL software’s histogram feature, three vascular indices were calculated from the adnexal tumors: vascularization index (VI), flow index (FI) and vascularization flow index (VFI). VI represents the number of vessels in the studied volume and is expressed as a percentage. FI reflects the intensity of blood flow at the time of the 3D sweep. VFI is interpreted to be a combination of VI and FI representing both vascularization and blood flow. FI and VFI are expressed as values ranging from 0 to 100 [12, 14, 15].

To estimate the interobserver agreement, another experienced ultrasound examiner (S.K.S.) re-evaluated the stored ultrasound data and classified the malignancy of tumors (expert opinion), and analysed 3D volumes.

The ultrasound examinations and off-line assessments were performed blinded for each other’s results and the histopathological diagnoses while analyzing the tumors. The preoperative findings were finally compared to postoperative histopathological diagnoses. For the purpose of this analysis, the borderline and low grade ovarian tumors were classified as malignant because normally also they are staged surgically. All participants gave their informed consent to the study, which was approved by the Ethics Committee of Tampere University Hospital (ETL R10080, 6 August 2010).

### Statistical analysis

No sample size calculation was performed due to the preliminary descriptive nature of this study. All data were analyzed using IBM SPSS Statistics for Windows, version 22 (Armonk, NY, IBM Corp.). The normality of the distributions of continuous variables was evaluated by Kolmogorov-Smirnov tests. Due to the skewness of the distributions, the comparisons of groups were performed using Mann-Whitney U-tests, and correlations were evaluated using Spearman’s correlation tests. Receiver operating characteristics curves (ROC) were used for evaluating the performance of serum CA125, RMI, LR2 and 3D vascular indices as predictive tests for malignancy. The best cut-off values of the models were calculated in consideration of sensitivity, specificity, positive predictive value and negative predictive value. Binomial variables were evaluated by Fischer’s exact tests or Pearson Chi-Square tests when appropriate.

The agreement of ultrasound features by both ultrasound examiners (R.J.N. and S.K.S.) was estimated by calculating the Cohen’s kappa index. A kappa value of less than 0.20 indicates poor agreement, 0.21–0.40 moderate agreement, 0.61–0.80 good agreement and 0.81–1.00 very good agreement [16]. Interobserver agreement of 3DPD indices were analyzed by related-samples Wilcoxon signed rank test.

## Results

In 100 patients with supposed adnexal masses, the tumors of 98 patients turned out to be true ovarian tumors, while the remaining two were appendiceal in origin. Of the 98 ovarian tumors, sixty-six (67.3%) were benign, of which 19 were bilateral. Respectively, 32 (32.7%) of the tumors were malignant, of which six were bilateral. Of the malignant tumors, 17 were epithelial serous, five epithelial mucinous, and eight of other pathology. Two of the malignant tumors were metastases of intestinal cancer. The histologic diagnoses of adnexal masses are presented in Table 1.

**Table 1:** Histology of the tumors

Histologic type	Number	Percent
Benign	66	(67.3)
Serous cystadenoma/adenofibroma	39	
Fibroma/thecoma	5	
Teratoma	7	
Brenner tumor	1	
Endometrioma	3	
Mucinous cystadenoma	3	
Serous/hemorrhagic cyst	7	
Bizarre leiomyoma	1	
Malignant	32	(32.7)
Epithelial tumors		
Serous		
High grade	9	
Low grade	3	
Borderline	5	
Mucinous		
Adenocarcinoma	3	
Borderline	2	
Clear cell carcinoma	1	
Carcinosarcoma	2	
Non-epithelial tumors		
Granulosa cell tumor	4	
Sertoli-Leydig cell tumor	1	
Metastatic^a^	2	
Total	98	(100.0)

^a^Primary tumors: sigmoid carcinoma and carcinoma of the appendix

The median age of the patients was 61 (range, 50–84) years. Tumor pathology did not depend on the age of the women or the bilaterality of the tumors. Three of the women in the benign ovarian tumor group and one in the malignant group were in fact perimenopausal, because they had experienced menstrual bleeding during the previous 6 months. Systemic hormone replacement therapy was used before surgery by 17 women with benign tumors and by five with malignant tumors. The median body mass index was 26 (range, 20–44) for women with benign tumors and respectively 27 (range, 19–39) for women with malignant tumors. The median diameters of the tumors in the groups were 6 (range, 3–11) and 7 (range, 3–12) cm, respectively. The median serum CA125 levels were 15 (range, 6–127) and 50 (range, 9–3195) kU/L, respectively, and the difference between the groups was significant (*p* < 0.001). The calculated median values of RMI for benign and malignant cases were 99 (range, 10–1143) and 360 (range, 27–7488), respectively (*p* < 0.001). The age of the patients, body mass indexes and CA125 levels did not correlate with each other.

The performances of the assessed methods are given in Table 2. Seventy-six (77.6%) of the tumors were classifiable using the simple rules, and 68 of these (89.5%) were correctly classified. Incorrectly classified tumors were all false-positive malignant tumors, and their histopathological diagnoses were serous cystadenoma/-fibroma (*n* = 4), thecoma (*n* = 2), one serous cyst and one Brenner tumor each. The unclassifiable tumors (*n* = 22) were evaluated by expert opinion (R.J.N.), and 77.3% (17 of 22) were classified correctly.

**Table 2:** Diagnostic performances of different methods at various cut-off values in detecting ovarian malignancy

Diagnostic method Cut-off value	Sensitivity %	95% CI	Specificity %	95% CI	PPV %	95% CI	NPV %	95% CI	Accuracy %
Serum CA125 (kU/L) → Area under the ROC curve = 0.80									
24	71.9	53.3–86.3	78.8	67.0–87.9	62.2	44.8–77.5	85.3	73.8–93.0	76.5
35	59.4	40.6–76.3	84.9	73.9–92.5	65.5	45.7–82.1	81.2	69.9–89.6	76.5
RMI → Area under the ROC curve = 0.81									
200	71.9	53.3–86.3	80.3	68.7–89.1	63.9	46.2–79.2	85.5	74.2–93.1	77.6
220	71.9	53.3–86.3	83.3	72.1–91.4	67.7	49.5–82.6	85.9	75.0–93.4	79.6
Simple rules with expert opinion^a^	90.6	75.0–98.0	84.6	73.9–92.5	74.4	57.9–87.0	94.9	85.9–98.9	86.7
Expert opinion	87.5	71.0–96.5	92.4	83.2–97.5	84.6	68.1–94.9	93.9	85.0–98.3	90.8
Tumor vascularity^b^	90.6	75.0–98.0	77.3	65.3–86.7	65.9	50.1–79.5	94.4	84.6–98.8	81.6
LR2 (%) → Area under the ROC curve = 0.93									
10	100.0	89.1–100.0	36.4	24.9–49.1	43.2	31.8–55.3	100.0	85.8–100.0	57.1
25	90.6	75.0–98.0	77.3	65.3–86.7	65.9	50.1–79.5	94.4	84.6–98.8	81.6
43	81.3	63.6–92.8	90.9	81.3–96.6	81.3	63.6–92.8	90.9	81.3–96.6	87.8

*IOTA* International Ovarian Tumor Analysis; *CI* Confidential interval; *PPV* Positive predictive value; *NPV* Negative predictive value; *ROC* Receiver operating characteristics; *RMI* Risk of malignancy index; *LR2* Logistic regression model 2

^a^Two-step strategy: as a result of inconclusive findings by simple rules, the expert evaluates the tumor

^b^Two-dimensional ultrasound vascular score 1-2 vs 3-4

The opinions of the experts (R.J.N. and S.K.S.) differed in five cases, including two serous and one mucinous cystadenoma, one teratoma, and one benign Brenner tumor. In addition, both experts failed in the case of two malignant tumors: one endometrioid adenocarcinoma FIGO Stage IA and one granulosa cell tumor. Two serous borderline cystadenomas were erroneously classified as benign. The interobserver agreement of the malignancy of tumors between both examiners was very good (Cohen’s kappa = 0.89, 95% CI 0.79–0.98; percentage of agreement of correct classification = 87.8%).

The tumor vascularity as examined by 2D ultrasound was higher in malignant tumors compared to benign tumors (*p* < 0.001). Predictions of tumor malignancy by scoring the vascularity yielded the same sensitivity (90.6%) as the simple rules with the expert opinion and LR2 with a cut-off value of 25. In comparison, 81.6% (80 of 98) of the tumors were classified correctly by vascularity assessment and LR2 (with a cut-off value 25), whereas the expert opinion correctly diagnosed 90.8% (89 of 98) of the cases. LR2 values and 3D vascular indices (VI, FI and VFI) were significantly different between benign and malignant tumors (*p* < 0.001). VI and VFI were the most sensitive and specific 3DPD indices (Table 3), being at least as specific as RMI, simple rules and LR2 in detecting malignant tumors. The subjective evaluation of tumor vascularity by 2DPD was more sensitive and almost as specific and accurate as 3DPD vascular indices. When 2D vascularity scoring or LR2 was combined to simple rules with the expert opinion, the specificity slightly increased, albeit at the expense of sensitivity (data not shown).

**Table 3:** Diagnostic performances of three-dimensional power Doppler indices at different cut-off values in detecting ovarian malignancy

3DPD indices Cut-off value	Sensitivity %	95% CI	Specificity %	95% CI	PPV %	95% CI	NPV %	95% CI	Accuracy %
VI (%) → Area under the ROC curve = 0.86									
1.00	67.7	48.6–83.3	90.9	81.3–96.6	77.8	57.7–91.4	85.7	75.3–92.9	83.5
FI → Area under the ROC curve = 0.72									
30	83.9	66.3–94.6	51.5	38.9–64.0	44.8	31.7–58.5	87.2	72.6–95.7	61.9
VFI → Area under the ROC curve = 0.87									
0.31	71.0	52.0–85.8	89.4	79.4–95.6	75.9	56.5–89.7	86.8	76.4–93.8	83.5

*3DPD* Three-dimensional power Doppler; *CI* Confidential interval; *PPV* Positive predictive value; *NPV* Negative predictive value; *ROC* Receiver operating characteristics; *VI* Vascularization index; *FI* Flow index; *VFI* Vascularization flow index

The interobserver agreement between the two experts was good also in the case of calculated 3DPD indices, as shown in Table 4.

**Table 4:** Interobserver agreement between two experts using three-dimensional power Doppler (3DPD) indices

3DPD indices	Expert opinion 1	Expert opinion 2	*p*-value^a^
	Median (25–75% quartiles)	Median (25–75% quartiles)	
VI	0.337 (0.069–1.098)	0.259 (0.066–0.894)	0.151
FI	31.796 (25.308–35.821)	31.693 (26.351–36.638)	0.839
VFI	0.112 (0.019–0.397)	0.079 (0.019–0.292)	0.257

*3DPD* Three-dimensional power Doppler; *VI* Vascularization index; *FI* Flow index; *VFI* Vascularization flow index

^a^Calculated by related-samples Wilcoxon signed rank test

By combining RMI and LR2 with VFI and using suitable cut-off values, the accuracies and specificities of RMI and LR2 improved, while their sensitivities decreased. On the other hand, combining these 3DPD indices with simple rules or the expert opinion did not improve either method’s accuracy. Newly created combination models are given in Table 5.

**Table 5:** Diagnostic performances of combined methods to detect malignancy at specific cut-off values

Developed method	Sensitivity %	95% CI	Specificity %	95% CI	PPV %	95% CI	NPV %	95% CI	Accuracy %
RMI > 200 and VFI > 0.31	51.6	33.1–69.9	95.5	87.3–99.1	84.2	60.4–96.6	80.8	70.3–88.8	81.4
Simple rules with expert opinion^a^ and VFI > 0.31	71.0	52.0–85.8	95.5	87.3–99.1	88.0	68.8–97.5	87.5	77.6–94.1	86.7
LR2 > 25 and VFI > 0.31	64.5	45.4–80.8	97.0	89.5–99.6	90.9	70.8–98.9	85.3	75.3–92.4	86.5

*IOTA* International Ovarian Tumor Analysis; *CI* Confidential interval; *PPV* Positive predictive value; *NPV* Negative predictive value; *RMI* Risk of malignancy index; *VFI* Vascularization flow index; *LR2* Logistic regression model 2

^a^Two step-strategy: As a result of inconclusive by simple rules, the expert evaluates the tumor

## Discussion

To the best of our knowledge, this is the first study to compare such a large spectrum of various preoperative methods (serum CA125, RMI, simple ultrasound rules, expert opinion, 2DPD and IOTA LR2) along with 3DPD indices for discrimination between benign and malignant ovarian tumors. The results of the present study imply that clinical expert opinion still provides the best method for diagnosing malignant ovarian tumors, with no additional benefit provided by use of 3DPD indices. Regarding the specificity of 3DPD indices, VI and VFI are only as specific as the traditional methods CA125 and RMI, but the combination of them with LR2 and RMI provides a clear improvement of specificity, but unfortunately at the expense of sensitivity.

The present results regarding the superiority of expert opinion are in accordance with previous studies. Timmerman et al. [5] found in their prospective study comparing IOTA-based simple rules, LR1 and LR2, and subjective assessment of the sonologist that expert’s opinion is either better or equivalent to the scoring systems.

A weakness of this study is the rather small number of patients, but on the other hand, all the patients were examined by the same investigator, which in turn can be considered to be an advantage. Moreover, the data was assessed by another examiner yielding a very good agreement between both examiners. Another advantage is a quite homogenous patient population, with only four perimenopausal women among 96 postmenopausal patients. A potential bias of the study is that although RMI was originally developed as a triage test for use by a less experienced ultrasound examiner to predict the malignancy of a tumor, in this study it was rather used by an ultrasound expert in parallel with simple rules and LR2 designed to triage patients who should be operated on by a gynecologic oncologist [5, 8]. The main rationale for including also RMI was to provide a reader less experienced in ultrasound, with a familiar comparator for the more specific triage methods.

CA125 is widely used as a tumor marker for epithelial ovarian cancer. It is quite accurate among postmenopausal women, while the relatively great number of false-positive results limits its utility in premenopausal setting. Our cut-off value was 35 kU/L, whereas some reports have used lower cut-offs, which might explain the lower sensitivity of CA125 (59.4%) in our study. In a review article on seven studies, the pooled sensitivity and specificity of CA125 in postmenopausal women were 85.9 and 85.2%, respectively [17]. We also tested a lower cut-off value of 24 kU/L, which clearly improved sensitivity, while somewhat weakening specificity.

In their original study, Jacobs et al. described a sensitivity of 85% and a specificity of 97% for a RMI cut-off level of 200 [1]. A review of RMI on 13 studies showed that for a cut-off level of 200, the pooled estimate for sensitivity was 78% (95% CI 71–85%) and for specificity was 87% (95% CI 83–91%), which is still better than in our study [18]. The reason for this may be our rather limited sample size, or the fact that the majority of the malignant tumors were of low malignant potential i.e. borderline epithelial, granulosa and Sertoli-Leydig cell tumors, which may have contributed to the less-than-optimal performance of RMI.

An IOTA-based protocol classified a woman as being at high risk for ovarian malignancy if the estimated LR2 risk was at least 25%, at intermediate risk if LR2 was between 5 and 25% and at low risk if LR2 was below 5% [19]. It has been presumed that the LR2-based protocol is more accurate than the RMI-based protocol, and is recommended to be used instead of RMI in discriminating ovarian tumors and concluding treatment protocols [19]. In the multicenter study of Testa et al., LR2 and RMI achieved AUC values of 0.90 and 0.85, respectively in 1,049 postmenopausal patients [6]. The corresponding AUC values in the present study were quite similar, 0.93 and 0.81. We argue that LR2 ≥ 25% risk is the most practical value in clinical work because in our study it achieved a negative predictive value of 94.4%.

Different preoperative scoring systems of ovarian tumors were evaluated in the meta-analysis of Kaijser et al. The pooled sensitivity and specificity of simple rules in five validated studies were found to be 93 and 81%, respectively [20]. However, the inconclusive cases were assessed to be malignant, which differs from our study where in the inconclusive results obtained by the simple rules protocol, the tumors were subjectively classified as being either benign or malignant by the same examiner. This is described as a two-step strategy [6]. In our data 85/98 of tumors were classified correctly by using this two-step strategy. Only three of 13 incorrectly classified tumors were false negative, or they were malignant although they were classified as benign. We reached equivalent results as the meta-analysis by Testa et al., which yielded a sensitivity of 93% and specificity of 83% for the two-step strategy in postmenopausal women [6]. Our results are also in line with a large database of IOTA studies which has shown that the pattern recognition by an experienced clinician is the best method in assessing ovarian tumors [8]. Recently, Piovano et al. showed that when serum CA125 was added to simple rules with subjective assessment for diagnosing ovarian tumors, the diagnostic accuracy increased, with an acceptable cost/benefit ratio [21].

In recent years, several studies have been published, where 3D ultrasound assessment has been used in gynecological cancers, including ovarian cancer. It has been shown that 3DPD vascular indices are elevated in malignant ovarian tumors [22]. Jokubkiene et al. also found this difference, but quantitative 3DPD indices of an ovarian tumor measured by another expert did not add any significant information to subjective quantitation by the original examiner of 2DPD findings [23]. Similarly, Guerriero et al. reported that 3DPD indices failed to improve accuracy as a secondary test for ovarian tumors alongside the evaluation of central vascularization by 2DPD [24]. On the contrary, Geomini et al. found that 3D ultrasonography significantly improved the discrimination of ovarian pathology, when they compared 2D to 3D ultrasound models [25]. Two methods of vascular sampling, manual [22] and spherical [23], have been purported to be alternatives to measuring the vascularity of the whole tumor, but based on our experience, the entire tumor should be taken account. In support of this, Kudla and Alcazar have shown that the size of the sphere volume does not affect power Doppler indices [26].

Our results do not differ from those of the only previous study that compared simple rules and 3DPD. The study of Silvestre et al. assessed 3DPD examination and simple rules in the identification of ovarian tumors [27]. They did not use other preoperative diagnostic methods, and the menopausal status of the patients was ignored. Similar to our study, the ovarian tumors were classified by conventional 2DPD according to IOTA vascular scores. They found that VI and VFI differentiated malignant tumors from benign ones, but they were not more accurate than 2D vascular scores. The use of 3D vascular indices did not decrease the number of false positive results obtained by simple rules.

## Conclusions

Combining the 3DPD vascular index VFI with RMI or LR2 may increase the specificity of the diagnostic test, but an ultrasound examination by an experienced clinician still seems to be the most reliable method in the preoperative work-up of ovarian tumors.

## Abbreviations

2D: Two-dimensional
2DPD: Two-dimensional power Doppler
3D: Three-dimensional
3DPD: Three-dimensional power Doppler
FI: Flow index
IOTA: International Ovarian Tumors Analysis
LR: Logistic regression model
RMI: Risk of malignancy index
VFI: Vascularization flow index
VI: Vascularization index
